# Recent Advances in Anti-virulence Therapeutic Strategies With a Focus on Dismantling Bacterial Membrane Microdomains, Toxin Neutralization, Quorum-Sensing Interference and Biofilm Inhibition

**DOI:** 10.3389/fcimb.2019.00074

**Published:** 2019-04-02

**Authors:** Osmel Fleitas Martínez, Marlon Henrique Cardoso, Suzana Meira Ribeiro, Octavio Luiz Franco

**Affiliations:** ^1^Programa de Pós-Graduação em Patologia Molecular, Faculdade de Medicina, Universidade de Brasília, Brasília, Brazil; ^2^Programa de Pós-Graduação em Ciências Genômicas e Biotecnologia, Centro de Análises Proteômicas e Bioquímicas, Universidade Católica de Brasília, Brasília, Brazil; ^3^S-inova Biotech, Programa de Pós-Graduação em Biotecnologia, Universidade Católica Dom Bosco, Campo Grande, Brazil; ^4^Programa de Pós-Graduação em Ciências da Saúde, Universidade Federal da Grande Dourados, Dourados, Brazil

**Keywords:** anti-virulence therapy, antibiotic resistance, bacterial membrane microdomains, quorum sensing, biofilms, bacterial toxins

## Abstract

Antimicrobial resistance constitutes one of the major challenges facing humanity in the Twenty-First century. The spread of resistant pathogens has been such that the possibility of returning to a pre-antibiotic era is real. In this scenario, innovative therapeutic strategies must be employed to restrict resistance. Among the innovative proposed strategies, anti-virulence therapy has been envisioned as a promising alternative for effective control of the emergence and spread of resistant pathogens. This review presents some of the anti-virulence strategies that are currently being developed, it will cover strategies focused on quench pathogen quorum sensing (QS) systems, disassemble of bacterial functional membrane microdomains (FMMs), disruption of biofilm formation and bacterial toxin neutralization.

## Introduction

Antimicrobial resistance has turned a serious concern to the human health, because in addition to the death caused by drug-resistant pathogens (~700,000 death annually and it is estimated ~10 million for the year 2050), important medical procedures such as organ transplantation, cancer chemotherapy and surgery are also compromised (O'Neill, 2016). Antimicrobial resistance is a multifactorial phenomenon. Therefore, to circumvent it, a range of actions are needed (WHO, 2018). According that, the innovative antimicrobial compounds development that operate under different principles to those of conventional antibiotics constitutes an important element in the battle against resistance (Munguia and Nizet, 2017). Among the new therapeutic strategies, anti-virulence therapy has emerged as a promising alternative since instead of killing the pathogens; it tries to deprive them from their virulence factors. Accordingly, the selective pressure exerted over pathogens should be lower than that exerted by conventional antibiotics and the emergence and spread of resistant mutants could be less frequent (Sully et al., 2014; Daly et al., 2015; Quave et al., 2015; Vale et al., 2016; Munguia and Nizet, 2017). However, *Pseudomonas aeruginosa* has developed resistance to anti-virulence drugs (Maeda et al., 2012; García-Contreras et al., 2013, 2015).

Virulence factors are microbial components (biomolecules and structures) used by pathogens to colonize, invade and persist in a susceptible host (Peterson, 1996; Defoirdt, 2017). The production of these factors is under the control of regulatory mechanisms; therefore, in principle interference with these regulatory mechanisms could affect the production of several virulence factors (Defoirdt, 2017). In this regard, quorum-sensing systems (QS) are involved in the regulation of the production of several virulence factors and consequently constitute one of the most exploited targets for the development of anti-virulence drugs (Defoirdt, 2017; Schütz and Empting, 2018). Moreover, the proper folding and/or oligomerization of virulence factors are pivotal for their biological activities. Therefore, the bacterial machinery involved in the virulence factors assembly is also a suitable target for disturbing pathogen virulence via anti-virulence drugs (Heras et al., 2015; Kahler et al., 2018). Recently, it has been described that bacterial functional membrane microdomains (FMMs) play a significant role in the assembly of several virulence factors, hence turning FMMs in an attractive target for drug development (García-Fernández et al., 2017; Koch et al., 2017; Mielich-Süss et al., 2017). In addition to disrupting the production and assembly of virulence factors; anti-virulence drugs have also been focused on interfering with the virulence factor functions (Mühlen and Dersch, 2016; Dickey et al., 2017). In that view, toxin neutralization constitutes a useful strategy to diminish the virulence of pathogens, as secretion of toxins is used by pathogens to colonize the host as well as to evade host immune system response (Heras et al., 2015; Kong et al., 2016; Rudkin et al., 2017). In addition, biofilm growing is a strategy used by pathogens to overcome the host immune system response (Gunn et al., 2016; Watters et al., 2016). Several anti-virulence strategies have been directed to disturb biofilm via interference with bacterial adhesion, extracellular matrix production or disintegration of existing biofilm (Feng et al., 2018; Liu et al., 2018; Puga et al., 2018).

**Figure 1:**
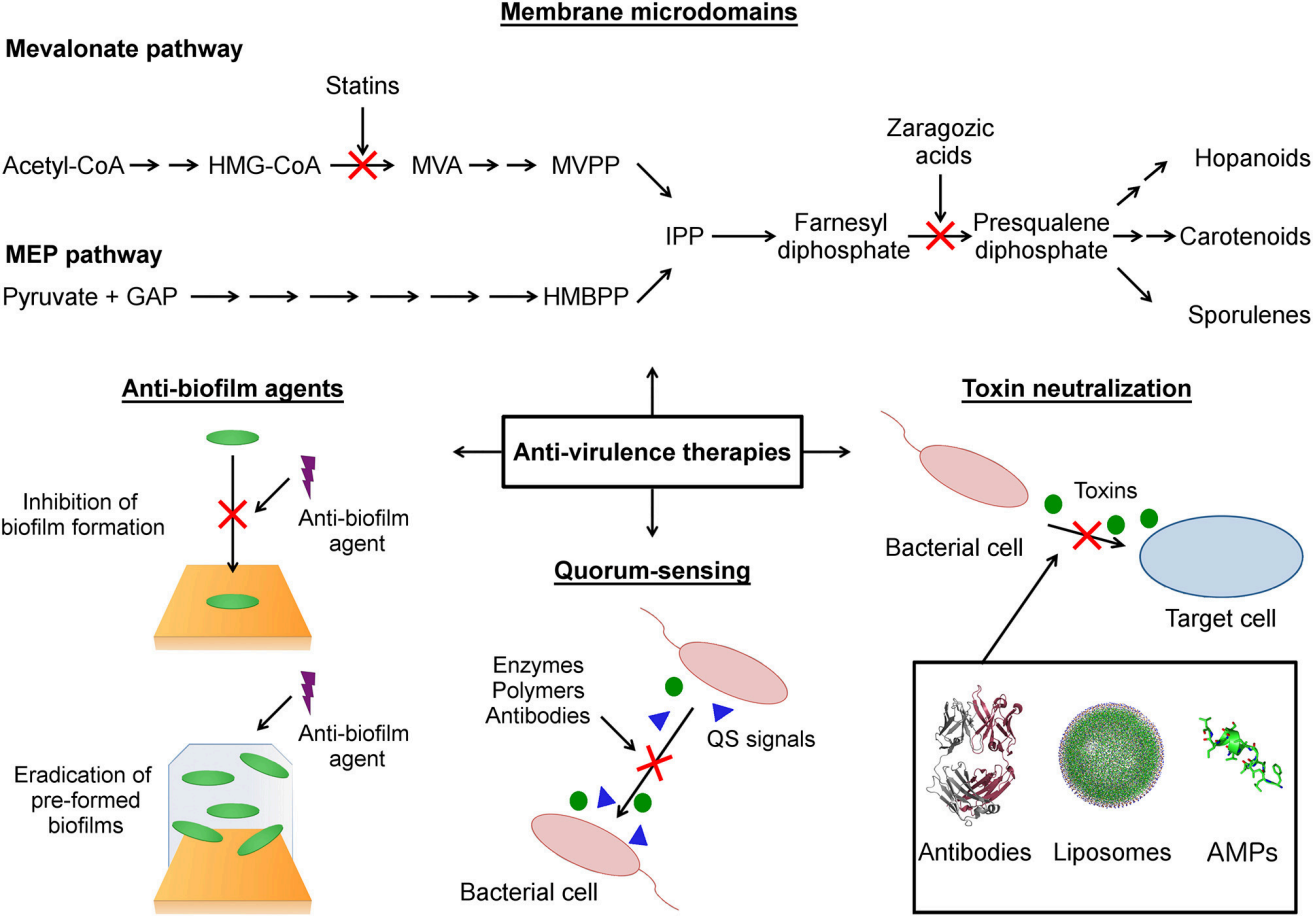
Schematic representation of anti-virulence strategies covered in this review. Membrane microdomains: The functional membrane microdomains (FMMs) are targeted by small molecules (statins, zaragozic acid) that inhibit the biosynthesis of their major constituent lipids (hopanoids, carotenoids). Anti-biofilm agents: This strategy focused on the use of agents that block the initial bacterial attachment to surface during biofilm formation and agents that destroy preformed biofilm. Quorum-sensing: The anti-virulence strategy that seeks modulate the production of virulence factors through interference with the quorum-sensing networks. Toxin neutralization: A strategy focused on block the action of toxins on host target cells. HMG-CoA (3-hydroxy-3-methylglutaryl-CoA), MVA (mevalonic acid), MVPP (5-diphosphomevalonate), GAP (D-glyceraldehyde-3-phosphate), HMBPP (4-hydroxy-3-methylbut-2-enyl-diphosphate), IPP (isopentenyl diphosphate), QS (quorum sensing), AMPs (antimicrobial peptides).

Given the significance attributed to anti-virulence therapy in the scientific community, and especially regarding antimicrobial resistance, this review is directed toward some recent findings in this area. It will uncover innovative strategies that are being implemented to quench pathogen quorum sensing (QS) systems, disassemble functional membrane microdomains (FMMs), disrupt biofilm formation and neutralize toxins (Figure 1 and Table 1). Some of the challenges that anti-virulence therapy faces as an emerging treatment in overcoming multidrug resistant pathogens will also be highlighted.

**Table 1:** Inhibitors of functional membrane microdomains assembly, quorum-sensing systems, biofilm formation, and toxin production and function.

**Inhibitor**	**Inhibitory activity**	**Bacteria**	**Virulence factors affected**	**References**
Zaragozic acid	Anti-FMM	*S. aureus*	•PBP2a oligomerization •Rny oligomerization •T7SS system assembly	García-Fernández et al., 2017; Koch et al., 2017; Mielich-Süss et al., 2017
Miltefosine	Anti-FMM	*S. aureus*	•Rny oligomerization	Koch et al., 2017
5-DSA	Anti-FMM	*S. aureus*	•Rny oligomerization •T7SS system assembly	Koch et al., 2017; Mielich-Süss et al., 2017
Simvastatin	Anti-FMM	*S. aureus*	•T7SS system assembly	Mielich-Süss et al., 2017
CRISPR-Cas9	Anti-QS Anti-biofilm	*E. coli* SE15	•Reduced biofilm formation •Down-regulation of *mqsR, pgaB, pgaC, csgE*, and *csgF*	Kang et al., 2017
CRISPR interference	Anti-QS Anti-biofilm	*E. coli* AK-117	•Reduced biofilm formation	Zuberi et al., 2017
2-(methylsulfonyl)-4-(1H-tetrazol-1-yl)pyrimidine	Anti-QS Anti-biofilm	*P. aeruginosa*	•Reduced biofilm formation •Reduced production of pyocyanin and pyoverdine	Thomann et al., 2016
(z)-5-octylidenethiazolidine-2, 4-dione (TZD-C8)	Anti-QS Anti-biofilm	*P. aeruginosa*	•Reduced biofilm formation •Reduced swarming motility	Lidor et al., 2015
Diketopiperazine	Anti-QS Anti-biofilm	*Burkholderia cenocepacia*	•Reduced biofilm formation •Reduced protease and siderophore production	Scoffone et al., 2016
ω-Hydroxyemodin	Anti-QS Anti-toxin	*S. aureus*	•Reduced RNAIII*, psmα* and *hla* transcription	Daly et al., 2015
Biaryl hydroxyketones	Anti-QS Anti-toxin	*S. aureus*	•Reduced RNAIII*, psmα* and *hla* transcription	Greenberg et al., 2018
(KFF)3 K peptide-conjugated locked nucleic acids	Anti*-* QS Anti-toxin	*S. aureus*	•Reduced expression of RNAIII, *psmα, psmβ, hla*, and *pvl*	Da et al., 2017
3-(2,4-dichlorophenyl)-1-(1H-pyrrol-2-yl)-2-propen-1-one	Anti-QS Anti-biofilm	*V. harveyi*	•Reduced biofilm production •Biofilm disintegration •Swimming and swarming motility reduction.	Rajamanikandan et al., 2017a
Zingerone	Anti-QS Anti-biofilm	*P. aeruginosa* PAO1*P. aeruginosa* clinical isolates.	•Reduced biofilm, pyocyanin, hemolysin, elastase, proteases, rhamnolipid production •Reduced swarming, swimming, and twitching motility	Kumar et al., 2015
AHL-nitric oxide hybrids	Anti-QS	*P. aeruginosa* PA14 *P. aeruginosa* PAO1	•Reduced pyocyanin and elastase production	Kutty et al., 2015
Flavonoids	Anti-QS	*P. aeruginosa* PA14	•Reduced pyocyanin production and swarming motility •*rhlA* transcription inhibition	Paczkowski et al., 2017
Terrein	Anti-QS Anti-biofilm	*P. aeruginosa* PAO1	•Reduced elastase, pyocyanin, rhamnolipid, and biofilm production •Attenuated *in vivo* virulence of *P. aeruginosa* PAO1 toward *C. elegans* and mice	Kim et al., 2018
Parthenolide	Anti-QS Anti-biofilm	*P. aeruginosa* PAO1	•Reduced pyocyanin, proteases, and biofilm production •Reduced swarming motility	Kalia et al., 2018
N-(4-{fluoroanilno}-butanoyl)-L-homoserine lactone N-(4-{chlororoanilno} butanoyl)-L-homoserine lactone	Anti-QS Anti-biofilm	*P. aeruginosa* PA330 *P. aeruginosa* PA282	•Reduced biofilm production Pyrone analogs	Anti-QS Anti-biofilm	*P. aeruginosa*	•Down–regulation of *lasA, lasB, rhlA, rhlB phzC1, and phzE1* •Reduced biofilm production	Park et al., 2015
Pyrone analogs	Anti-QS Anti-biofilm	*P. aeruginosa*	•Down–regulation of *lasA, lasB, rhlA, rhlB phzC1, and phzE1* •Reduced biofilm production	Park et al., 2015
Pyridoxal lactohydrazone	Anti-QS Anti-biofilm	*P. aeruginosa* PAO1	•Reduced biofilm, alginate and pyocyanin production •Reduced swarming and twitching motility	Heidari et al., 2017
1,5-dihydropyrrol-2-ones analogs	Anti-QS	*E. coli* JB357 *gfp* reporter strain	•QS inhibition	Goh et al., 2015
Triaryl derivatives	Anti-QS	*E. coli* BL21 DE3 Gold reporter strain		Capilato et al., 2017
Triphenyl scaffold-based hybrid compounds	Anti-QS	*E. coli* JLD 271 reporter strain		O'Reilly and Blackwell, 2015
Non-native AHL	Anti-QS	*E. coli* JLD 271 and *P. aeruginosa* PAO-JP2 reporter strains		Eibergen et al., 2015
Fluoro-substituted Isothiocyanates	Anti-QS	*P. aeruginosa*	•Reduced pyocyanin production •Reduced swarming motility •Attenuated *in vivo* virulence of *P. aeruginosa* PAO1-UW toward *C. elegans* •Attenuated *P. aeruginosa* PA14 virulence in an *ex-vivo* human skin burn wound model	Amara et al., 2016
Zeaxanthin	Anti-QS Anti-biofilm	*P. aeruginosa* PAO1	•Reduced biofilm formation •Downregulated *rhlA* and *lasB* expression	Gökalsin et al., 2017
Phenyllactic acid	Anti-QS Anti-biofilm Anti-toxin	*P.aeruginosa* PAO1 and clinical isolates	•Reduced pyocyanin, proteases, rhamnolipid, and hemolysin production •Reduced swarming motility •Reduced biofilm production	Chatterjee et al., 2017
Metformin	Anti-QS Anti-biofilm Anti-toxin	*P. aeruginosa* PAO1	•Reduced biofilm, pyocyanin, proteases, hemolysin and elastase production •Reduced swimming and twitching motility	Abbas et al., 2017
Glyceryl trinitrate	Anti-QS Anti-biofilm	*P. aeruginosa* PAO1 and clinical isolates	•Reduced biofilm, pyocyanin and proteases production	Abbas and Shaldam, 2016
4-amino-quinolone-based compounds	Anti-QS Anti-biofilm	P. aeruginosa PAO1-L P. aeruginosa PA14	•Reduced biofilm and pyocyanin production	Soukarieh et al., 2018
Lactam-bridged AIP analogs	Anti-QS	*S. aureus* reporter strains		Tal-Gan et al., 2016
Solonamides analogs	Anti-QS Anti-toxin	*S. aureus*	•Reduced *RNAIII* and *hla* expression •Marginally enhanced biofilm formation	Baldry et al., 2016
AIP-II peptidomimetics	Anti-QS	*S. aureus* reporter strains		Vasquez et al., 2017
Lactam hybrids of solonamide B and AIP	Anti-QS	*S. aureus* RN10829 reporter strain		Hansen et al., 2018
Truncated AIP	Anti-QS	*S. lugdunensis* AH4031 reporter strain		Gordon et al., 2016
AIP analogs	Anti-QS Anti-biofilm	*S. epidermidis* RP62A	•Reduced biofilm formation (using non-native agonist of AgrC-type I)	Yang et al., 2016
Linear peptidomimetics	Anti-QS Anti-toxin	*S. aureus* 8325-4 *S. aureus* reporter strains	•Reduced expression of RNAIII •Reduced *hla* expression	Karathanasi et al., 2018
Bicyclo [2.2.1] hept-5-ene-2,3-dicarboxylic acid 2,6-dimethylpyridine 1-oxide	Anti-QS Anti-biofilm	*V. harveyi*	•Reduced biofilm production •Disintegrated mature biofilm •Reduced swarming and swimming Coumarin	Anti-QS Anti-biofilm	*P. aeruginosa* PAO1 and clinical isolates	•Reduced biofilm production •Down-regulation of *lasI, rhlI,rhlR,pqsB, pqsC, pqsH, ambBCDE* •Reduced protease and pyocyanin production •Reduced expression of T3SS secretion system-associated genes	Zhang et al., 2018
Coumarin	Anti-QS Anti-biofilm	*P. aeruginosa* PAO1 and clinical isolates	•Reduced biofilm production •Down-regulation of *lasI, rhlI,rhlR,pqsB, pqsC, pqsH, ambBCDE* •Reduced protease and pyocyanin production •Reduced expression of T3SS secretion system-associated genes	Zhang et al., 2018
T315 compound	Anti-biofilm	*S. enterica* serovar Typhimurium *S*. *enterica* serovar Typhi *A. baumannii*	•Reduced biofilm production	Moshiri et al., 2018
2-aminobenzimidazole derivatives	Anti-biofilm	*S. enterica* serovar Typhimurium	•Reduced biofilm production	Huggins et al., 2018
[3-(2-furylmethyl)-2-[[(5-hydroxy-1H-pyrazol-3-yl)methyl]thio]-3,5,6,7-tetrahydro-4H-cyclopenta [4,5]thieno[2,3-*d*]pyrimidin-4-on]	Anti-biofilm	*S. enterica* serovar Typhi *S. enterica* serovar Typhimurium *A. baumannii*	•Reduced biofilm production	Koopman et al., 2015
3F1 compound	Anti-biofilm	*S. mutans*	•Biofilm dispersion	Garcia et al., 2017
2-amino-imidazole/triazole conjugate	Anti-biofilm	*S. mutans*	•Reduced biofilm production	Pan et al., 2015b
Peptidomimetic compounds	Anti-biofilm	*Porphyromonas gingivalis*	•Three-species biofilm inhibition	Tan et al., 2018
1,2,3-triazole-based peptidomimetics	Anti-biofilm	*P. gingivalis*	•Reduced two-species biofilm formation (inhibition of adherence of *P. gingivalis* to *S. gordonii*)	Patil et al., 2016
Kaempferol	Anti-biofilm	*S. aureus*	•Reduced biofilm production (inhibition of initial attachment) •Inhibition of sortase A activity •Downregulation of *clfA, clfB, fnbA* and *fnbB* expression	Ming et al., 2017
5-benzylidene-4-oxazolidinones	Anti-biofilm	*S. aureus*	•Reduced biofilm production •Biofilm dispersion	Edwards et al., 2017
p-tolyl(3-phenylpropyl)carbamate	Anti-biofilm	*S. aureus*	•Reduced biofilm production	Stephens et al., 2016
Antibiofilm compound 1 (ABC-1)	Anti-biofilm	*S. aureus*	•Reduced biofilm production •Reduced polysaccharide intercellular adhesin (PIA) production and eDNA release •Downregulation of *spa* expression	Shrestha et al., 2016
Zosteric acid derivatives	Anti-biofilm	*E. coli*	•Reduced biofilm production	Cattò et al., 2015
Pyrimidinedione	Anti-biofilm	*S. pneumoniae* *S. aureus* *S. epidermidis*	•Reduced biofilm production	Yadav et al., 2015
Resveratrol	Anti-toxin Anti-QS	*S. aureus*	•Downregulation of *hla, RNAIII* and *saeRS* expression •Reduced α-hemolysin production	Duan et al., 2018; Tang et al., 2018
Lysionotin	Anti-toxin Anti-QS	*S. aureus*	•Downregulation of *hla*, and *agr* expression •Reduced α-hemolysin production	Teng et al., 2017
Eriodictyol	Anti-toxin Anti-QS	*S. aureus*	•Downregulation of *hla* and *RNAIII* expression •Reduced α-hemolysin production •Reduced hemolysis	Xuewen et al., 2018
Chalcone	Anti-toxin Anti-QS Anti-biofilm	*S. aureus*	•Downregulation of *hla* and *agrA* expression •Reduced α-hemolysin production •Inhibition of Sortase A activity •Reduced adherence to fibronectin •Reduced hemolysis •Reduced biofilm formation Prim-O-Glucosylcimifugin	Anti-toxin Anti-QS	*S. aureus*	•Reduced α-hemolysin production •Downregulation of *hla* and *RNAIII* expression •Reduced hemolysis	Ping et al., 2018
Prim-O-Glucosylcimifugin	Anti-toxin Anti-QS	*S. aureus*	•Reduced α-hemolysin production •Downregulation of *hla* and *RNAIII* expression •Reduced hemolysis	Ping et al., 2018
Dracorhodin perochlorate	Anti-toxin Anti-QS	*S. aureus*	•Reduced α-hemolysin production •Downregulation of *hla* and *RNAIII* expression •Reduced hemolysis	Liu et al., 2017
Sclareol	Anti-toxin Anti-QS	*S. aureus*	•Reduced α-hemolysin production •Downregulation of *hla* and *RNAIII* expression •Reduced hemolysis	Ping et al., 2017
2-aminoimidazole derivatives	Anti-toxin	*Clostridium difficile*	•Reduced toxin activity	Thanissery et al., 2018
Peptides	Anti-toxin	*Aggregatibacter actynomycetemcomitans*	•Inhibition of LtxA-mediated cytotoxicity	Krueger et al., 2018
Galloylated catechins	Anti-toxin	*A. actynomycetemcomitans*	•Inhibition of LtxA-mediated cytotoxicity	Chang et al., 2019

## Dismantling Bacterial Membrane Microdomains

To develop new therapeutic strategies against multidrug-resistant pathogens, a suitable approach could be designing antimicrobial compounds that target bacterial structures other than the targets of the major conventional antibiotics. In this respect, the bacterial cytoplasmic membrane constitutes an attractive target as it functions as a barrier that maintains favorable intracellular physicochemical conditions for the correct development of bacterial metabolism. In addition, the cytoplasmic membrane regulates the exchange of information and substances with the extracellular medium (Poolman et al., 2004; Strahl and Errington, 2017). Structural changes in membranes to resist antimicrobial compounds could involve an elevate fitness cost and consequently could be less likely to occur (Zasloff, 2002). However, it has been reported membrane modifications linked to resistance toward antimicrobial compounds (Nuri et al., 2015; Joo et al., 2016). Therefore, compounds that interfere with structural organization and/or functions associated with membranes without affecting bacterial growth could be desirable.

In particular, bacterial membranes contain FMMs which are eukaryotic lipid-raft–like domains that enclose a characteristic lipid and protein composition. Specifically, they appear be rich in polyisoprenoid lipids like hopanoids and carotenoids, conferring compact, rigid, and hydrophobic features that could limit the diffusion of FMM-associated proteins away from them (Bramkamp and Lopez, 2015). As regards FMM-associated protein composition, bacterial protein flotillins are essential FMM components. These proteins are closely associated with microdomains and are involved in the membrane fluidity regulation as well as promoting and stabilizing the assembly of specific protein complexes via their scaffold activity (Lopez and Koch, 2017). Other FMM-associated proteins are involved in signaling networks (e.g., sensor kinase KinC), protein secretion machinery (e.g., Sec Y and Sec A) and proteolytic complexes (e.g., FtsH protease complex) (Bramkamp and Lopez, 2015; Lopez and Koch, 2017). Most of these FMM-associated proteins are functionally active when they form multimeric complexes. Therefore, FMMs could be seen as protein complexes assembly platforms with punctuating distribution along bacterial cytoplasmic membrane, where the recruited proteins undergo efficient oligomerization and consequently become functionally active (Bramkamp and Lopez, 2015; Lopez and Koch, 2017).

Experimental evidence has shown that mutant strains in genes involved in the production of FMM structural components could be hampered in establishing virulence determinants. A *Bacillus subtilis* mutant in the *yisP* gene was ineffective in biofilm formation. The gene *yisP* encodes for phosphatase YisP, which produces farnesol from farnesyl diphosphate (López and Kolter, 2010; Feng et al., 2014). Moreover, *B. subtilis* double mutant (Δ*floT* Δ*yqfA*) in genes that encode for the flotillin-like proteins FloT and YqfA (FloA) was defective in the formation of biofilms and sporulation (López and Kolter, 2010; Yepes et al., 2012). *Bacillus subtilis* mutants Δ *yisP*, Δ *yuaG*, Δ *yqfA*, and Δ *yuaG* Δ *yqfA* showed diminished Sec-dependent secretion efficiency (Bach and Bramkamp, 2013). Furthermore, a *Campylobacter jejuni* mutant in the gene *cj0268c* displayed reduced adherence to Caco2 cells. The gene *cj0268c* encodes for a protein that contains a SPFH domain, which is typical for flotillin-like proteins (Tareen et al., 2013). In addition, gnotobiotic IL-10^−/−^ mice infected with *C. jejuni cj0268c* mutant developed reduced intestinal immunopathology in comparison with ones infected with parental strain and complemented strain (Heimesaat et al., 2014). Recently, it was observed that lack of the gene *floA* in *Staphylococcus aureus* may impact the function of the type VII secretion system (T7SS). Consequently, the Δ*floA* mutant exhibited a reduced virulence in a murine model (Mielich-Süss et al., 2017). Similarly, another study showed that the *S. aureus* Δ*floA* mutant exhibited diminished virulence in both an invertebrate infection model (*Galleria mellonella*) and a murine infection model. Specifically, a perturbed degradosome activity was observed; probably by the defective FloA-assisted oligomerization of RNase Rny in the Δ*floA* mutant (Koch et al., 2017). All these experimental items of evidence suggest that FMMs could be an attractive target to develop anti-virulence therapy.

In accordance with the above described experimental evidence, several studies have demonstrated that small molecules, which interfere with polyisoprenoid lipid biosynthesis metabolic pathways and with FMM′ physicochemical properties, attenuated the virulence of pathogens *in vitro* and *in vivo* (Table 1) (García-Fernández et al., 2017; Koch et al., 2017; Mielich-Süss et al., 2017). Isoprenoids are organic molecules that involve significant diversity of chemical structures and functions; however, they all derive from isopentyl diphosphate (IPP) or its isomer dimethylallyl diphosphate (DMAPP) (Heuston et al., 2012; Pérez-Gil and Rodríguez-Concepción, 2013). These precursor molecules are synthesized in bacteria through two metabolic pathways including the mevalonate pathway and the 2C-methyl-_D_-erythritol 4-phosphate (MEP) pathway. The MEP pathway is used by most bacteria. However, the mevalonate pathway is used exclusively by pathogens like *Borrelia burgdorferi, S. aureus, Streptococcus pneumoniae, Enterococcus faecalis*, and is also present in animals (Wilding et al., 2000a; Pérez-Gil and Rodríguez-Concepción, 2013). Moreover, *Listeria monocytogenes* and some *Streptomyces* strains contain both pathways. Bacteria such as *Rickettsia* and *Mycoplasma* do not contain genes that encode for the enzymes involved in the mevalonate and MEP pathways, and therefore they lack both isoprenoid pathways (Pérez-Gil and Rodríguez-Concepción, 2013).

Because the mevalonate pathway is shared between humans and bacteria, in principle, hypercholesterolemia-treating drugs in humans could exert an inhibitory effect on the bacterial mevalonate pathway (Bramkamp and Lopez, 2015). Statins are a class of drugs that target the enzyme class I HMG-CoA reductase (3-hydroxy-3-methylglutaryl-Coenzyme A reductase), which catalyzes the conversion of 3-hydroxy-3-methylglutaryl-Coenzyme A (HMG-CoA) to coenzyme A (CoA) and mevalonate via reductive deacylation, in the human mevalonate pathway (Istvan and Deisenhofer, 2001; Tobert, 2003). Statins bind to the class I HMG-CoA reductase active site, affecting the binding of biological substrate HMG-CoA, and therefore act as competitive inhibitors (Istvan and Deisenhofer, 2001). Similarly, as takes place for human HMG-CoA reductase, statins are also class II HMG-CoA reductase (bacteria and archaea) inhibitors. However, statins exert a diminished inhibitory activity toward class II HMG-CoA reductases [inhibition constant (Ki) in the micro and millimolar range] than class I (Ki in the nanomolar range), which appear to be associated with structural differences between these enzyme classes (Alberts et al., 1980; Kim et al., 2000; Wilding et al., 2000b; Tabernero et al., 2003; Hedl and Rodwell, 2004; Haines et al., 2013). It is important to highlight that some statins with antibacterial activity against Gram-positive and Gram-negative bacteria have been reported. Such capability matched to their wide clinical use, potentially favoring the statins resistance emergence (Ko et al., 2017).

In addition to statins, another inhibitors of bacterial HMG-CoA reductase are the plant-derived compounds annonaceous acetogenins. Feng et al. (2011) showed that squamostating A, squamostating B, squamocin C, and asimicin A were more potent *S. pneumoniae* HMG-CoA reductase inhibitors than lovastatin (Feng et al., 2011). Moreover, Li et al. (2012) using a structure-based screening approach identified several potential *S. pneumoniae* HMG-CoA reductase inhibitors with IC_50_ values in the μM range (Li et al., 2012).

Other enzymes that have been targeted to disrupt the production of polyisoprenoids are the bacterial “head-to-head” terpene synthases. This class of enzymes catalyzes the formation of a cyclopropylcarbinyl diphosphate intermediate (e.g., presqualene diphosphate) via C1′-2, 3 condensation of two isoprenoid diphosphate molecules (e.g., farnesyl diphosphate) (Lin et al., 2010). Subsequently, the presqualene diphosphate undergoes rearrangement that involves ring opening catalyzed by the same “head-to-head” terpene synthase [e.g., *S. aureus* dehydrosqualene synthase (CrtM)] or by partner enzymes (e.g., *Zymomonas mobilis* HpnC) rendering dehydrosqualene or hydroxysqualene (Lin et al., 2010; Pan et al., 2015a; Schwalen et al., 2017). Because the “head-to-head” terpene synthases act on IPP synthesis downstream, inhibitors as zaragozic acid family compounds, which are potent inhibitors of “head-to-head” terpene synthases, could hinder the production of polyisoprenoid lipids in many bacterial species and, therefore, disturb the structural assembly and function of the FMMs (Bramkamp and Lopez, 2015).

Supporting the potential use of these sterol synthesis inhibitory drugs as effective anti-FMM drugs, García-Fernández et al. (2017) showed that the penicillin-binding protein PBP2a, which mediates resistance to β-lactam antibiotics in the pathogen methicillin-resistant *Staphylococcus aureus* (MRSA), was associated with FMMs. The treatment of the MRSA culture with 50 μM of zaragozic acid did not affect the bacterial growth but affected FMMs formation with the consequent disturbance of PBP2a oligomerization, which could imply a non-optimal functionality of PBP2a. In support of this, zaragozic acid-treated MRSA showed a β-lactam-sensitive phenotype in comparison with the non-treated MRSA. Likewise, MRSA treatment with simvastatin, lovastatin, mevastatin and pravastatin enhanced the antimicrobial activity of oxacillin toward the resistant pathogen. Moreover, oxacillin-treated MRSA-infected mice showed a significantly lower survival rate than oxacillin/zaragozic acid-treated MRSA-infected mice. Mice infected with a MRSA strain isolated from a pneumonia patient and treated with oxacillin exhibited a significantly higher bacterial load in lungs than oxacillin/zaragozic acid-treated mice (García-Fernández et al., 2017). This study show the potential of anti-FMM drugs to revert an antibiotic-resistant phenotype into an antibiotic-sensitive phenotype. Besides, it has been demonstrated that treatment of *S. aureus* with zaragozic acid, miltefosine and 5-doxyl-stearic acid (5-DSA) (miltefosine and 5-DSA alter physicochemical properties of lipid-rafts) disturbed FMMs assembly and consequently inhibited FloA oligomerization and scaffold activity. Impaired functional FloA could yield non-proper oligomerization of the RNase Rny, therefore affecting the *S. aureus* degradosome machinery, which is important for the regulation of virulence factors-coding genes expression (Koch et al., 2017). Using a murine infection model it was observed that *S. aureus-* infected mice treated with zaragozic acid, miltefosine, or 5-DSA displayed a significantly higher survival rate than non-treated infected mice. Additionally, in another infection experiment, it was demonstrated that in the treated animals' lungs, small RNAs *rsaA* and *sau63* had significantly higher expression in comparison to untreated controls. This suggested defective degradosome function mediated by the inhibition of FloA oligomerization *in vivo* (Koch et al., 2017). Other evidence of anti-FMM drugs' potential in anti-virulence therapy was recently revealed. It was observed that *S. aureus* treatment with zaragozic acid, simvastatin, or 5-DSA disturbed the correct assembly of T7SS and consequently affected the secretion of T7SS-associated virulence factors. It was suggested that this effect of the anti-FMM compounds on T7SS functionality could involve impaired FloA scaffold activity, since FloA interacted with the component EssB of T7SS and assisted in the complex assembly. Additionally, it was verified that in BALB/c mice infected with *S. aureus* and treated with zaragozic acid the levels of IgM antibodies against T7SS substrates such as Esx A Esx B, Esx C, and Esx D were inferior to those in non-treated controls (Mielich-Süss et al., 2017).

## Manipulating Bacterial Quorum Sensing Systems

For the establishment of successful host infection by pathogenic bacteria it is necessary to have coordinated actions among the population members of the infecting pathogen. These synchronized actions may be achieved through communication systems between bacteria. Therefore, bacterial communication systems are important players in the establishment of a successful host-infection process and consequently are attractive targets for developing anti-virulence therapeutic strategies (Defoirdt, 2017; Munguia and Nizet, 2017). One of the bacterial communication systems that is most studied and distributed among bacteria is the quorum-sensing (QS) network. Independently of the diversity of bacterial QS systems' architecture and functional components, these communication systems are commonly based on a sequence of events that consist of the production of chemical signaling molecules (autoinducers), which are secreted to the external medium and accumulate until reaching a threshold of concentration that is detected by bacteria. Subsequently, a change in the gene-expression patterns take place in response to the detected signaling molecules (Waters and Bassler, 2005; Papenfort and Bassler, 2016). The strategies for disturbing and manipulating QS networks aim to interfere with these basic events (Table 1).

However, these quorum quenching strategies have to face several challenges to become feasible therapeutic options. The expression of certain virulence factor could be subjected to the control of several regulatory mechanisms other than the targeted QS system (Arya and Princy, 2016). Depending on the environment factors find by the pathogen; some of these regulatory mechanisms could influence the virulence factor expression more predominantly than others (Goerke et al., 2001; Xiong et al., 2006; Zurek et al., 2014; Liu et al., 2016). Another element that could represent a challenge is the diversity of QS systems that could be present in a pathogen; and that these could form complex interconnected networks (Lee and Zhang, 2015; Koul et al., 2016). Furthermore, in some cases, the interference with the QS systems could promote the virulence instead of attenuated it (Köhler et al., 2010; García-Contreras, 2016). In addition, interference with QS systems could affect the pathogen growth, which could exert selective pressure and facilitate the emergence of resistant pathogens (García-Contreras, 2016). Moreover, in a polymicrobial infection, because the interaction between pathogens could be mediate by QS-controlled factors, the interference with QS systems in a pathogen potentially could facilitate the pathogenicity and antibiotic resistance of the co-infecting pathogens (O'brien and Fothergill, 2017; Radlinski et al., 2017). Also, it is important to have diagnostic tools sensitive enough that allow the detection of the infecting pathogen at low cellular densities; so that quorum quenching strategies could be implemented before the pathogen reach the quorum necessary to trigger their pathogenic potential (Kalia et al., 2019).

### Interference With Quorum-Sensing Signal Biosynthesis

One of the approaches to disrupting QS systems is based on the interference in signal production. This strategy is centered on inhibiting the activity of the autoinducer-producing enzymes via small inhibitory molecules, which are mainly substrate structural analogs or transition state analogs and do not affect bacterial growth (LaSarre and Federle, 2013). The advantages of this strategy are that enzymes involved in the production of autoinducers are not present in mammalian cells and are encoded in the genome of several bacterial species (LaSarre and Federle, 2013; Pereira et al., 2013). Moreover, it is possible that an effect on the activity of a particular enzyme [e.g., 5′-methylthioadenosine/S adenosylhomocysteine nucleosidase (MTAN nucleosidase)] could in turn affect the production of more than one type of autoinducer (Gutierrez et al., 2009). However, the fact that target enzymes have intracellular localization raises several challenges for the implementation of this strategy. Primarily, the inhibitory compounds should overcome the diffusion barriers imposed by bacterial surface structures, particularly difficult in Gram-negative bacteria, which contain a double membrane system. Furthermore, once inside the cell, the inhibitors could be expelled to extracellular space by efflux pumps. In addition, the inhibitor compounds could inhibit enzymes involved in important metabolic processes, compromising the cellular viability, and therefore creating selective pressure on the bacteria (Hinsberger et al., 2014; Sahner et al., 2015; Ji et al., 2016).

Despite all these possible limitations, several studies using *in cellulo* and *in vivo* approaches suggested the feasibility of the use of autoinducer-producing enzyme inhibitors. Gutierrez et al. (2009) showed that transition state analogs of the enzyme MTAN nucleosidase suppressed the production of autoinducers by *Vibrio cholerae* and enterohemorrhagic *Escherichia coli* O157:H7. Specifically, the inhibitors 5′-methylthio-DADMe-Immucillin-A, 5′-ethylthio-DADMe-Immucillin-A, and 5′-butylthio-DADMe-Immucillin-A inhibited the MTAN activity in a dose-dependent fashion, and consequently the production of autoinducers, without disturbing bacterial growth in *V. cholerae* N1696 culture. Similar behavior was observed in *Escherichia coli* O157:H7 culture, where 5′-methylthio-DADMe-Immucillin-A and 5′-butylthio-DADMe-Immucillin-A inhibited the production of AI-2 in a dose-dependent mode, without affecting bacterial growth. Importantly, both bacterial strains maintained the 5′-butylthio-DADMe-Immucillin-A sensitive phenotype after growth for several generations when challenged with the inhibitor, suggesting that there was no emergence of resistance. In agreement with the reduction of AI-2 production, reduced biofilm-forming capacity was observed in both bacterial species when they were treated with the 5′-butylthio-DADMe-Immucillin-A inhibitor (Gutierrez et al., 2009).

Another bacterial pathogen for which QS signal-producing enzyme inhibitors have been developed is *Pseudomonas aeruginosa*. This pathogen contains a particular QS system [*Pseudomonas* quinolone system (*pqs*)] which uses PQS (3,4-dihydroxy-2-heptylquinoline, *Pseudomonas* quinolone signal) and HHQ (2-heptyl-4-hydroxyquinoline) as QS signal molecules (LaSarre and Federle, 2013; Papenfort and Bassler, 2016). The production of HHQ and PQS starts with activity of the enzyme anthranilyl-CoA ligase (PqsA) which catalyzes the activation of anthranilate to anthraniloyl-coenzyme A via an anthranilyl-AMP reaction intermediate. Sulfonyladenosine compounds (anthranilyl-MAS and anthranilyl-AMSN) that mimic the anthranilyl-AMP intermediate were PqsA inhibitors that reduced the production of HHQ and PQS by *P. aeruginosa* PA14 strain (Ji et al., 2016). In addition to intermediate-mimic compounds, other PqsA inhibitors are substrate structural analogs. *P. aeruginosa* PAO1strain challenged with the anthranilate analog methyl-anthranilate reduced in a dose-dependent fashion PQS production as well as elastase activity. Elastase is a virulence factor that is under the control of the *pqs* QS system (Calfee et al., 2001). However, in other study, the effect of methyl-anthranilate in reducing *P. aeruginosa* PA14-produced PQS and HHQ levels was less potent, and the most powerful compounds were halogenated anthranilate analogs. In addition, some of these halogenated analogs showed effectivity *in vivo* in reducing the virulence and limiting *P. aeruginosa* systemic dissemination in infected mice (Lesic et al., 2007). In addition to PqsA, another enzyme linked to the PQS biosynthesis pathway that has been targeted is PqsD. This enzyme catalyzes the formation of the 2-aminobenzoylacetyl-CoA by the condensation of anthraniloyl-CoA with malonyl-CoA via a tetrahedral transition state. The treatment of *P. aeruginosa* PA14 with the potent PqsD inhibitor (2-nitrophenyl) phenyl methanol disturbed the production of HHQ and PQS as well as reducing the biofilm volume (Storz et al., 2012). Moreover, some catechol-derivative compounds that act as PqsD inhibitors by blocking the access of the natural substrate to the active site of PqsD reduced the production of HHQ by *P. aeruginosa* (Allegretta et al., 2015).

Furthermore, the enzyme PqsBC, which catalyzes the condensation of octanoyl-CoA and 2-aminobenzoylacetate rendering HHQ is also a QS inhibitor target. In this regard, it was observed that the PqsBC competitive inhibitor 2-aminoacetophenone (2-AA) affected HHQ production by the recombinant *P. putida* KT2440 strain (Drees et al., 2016). However, strategies focused on PqsBC inhibition could carry unwanted effects. The PqsBC substrate 2-aminobenzoylacetate could transform in 2-AA or 2,4-dihydroxyquinoline (DHQ) (Dulcey et al., 2013). The 2-AA promoted the emergence of persister cells in pathogens as *P. aeruginosa, A. baumannii* and *Burkholderia thailandensis*, whereas DHQ has been linked to *P. aeruginosa* pathogenicity (Que et al., 2013; Gruber et al., 2016). In principle, the inhibition PqsBC could provoke the accumulation of 2-aminobenzoylacetate, favoring the formation of 2-AA and DHQ, which could promote the emergence of antibiotic tolerant pathogens and/or increase in the pathogenicity (Allegretta et al., 2017; Maura et al., 2017). In this regard, it has been demonstrated that the treatment of a *P. aeruginosa mvfR* mutant (constitutively expressed the *pqs ABCDE* operon) with some benzamide-benzimidazole compounds (PqsBC inhibitors) may provoked the accumulation of 2-AA and DHQ (Maura et al., 2017). In addition, in the *P. aeruginosa* PA14 parental strain, the treatment with some of these PqsBC inhibitors (specifically those also contain a low anti-MvfR (PqsR) activity) did affect only partially the production of 2-AA and DHQ and did not inhibit the tolerance to meropenem (Maura et al., 2017). In another study performed by Allegretta et al. (2017), the treatment of *P. aeruginosa* PA14 and a PA 14 *pqsH* mutant with PqsBC inhibitors, produced an increase at 2-AA and DHQ levels. One of these PqsBC inhibitors increased the subpopulation of persister *P. aeruginosa* PA14 cells to levels similar to a *pqsBC* mutant strain (Allegretta et al., 2017). Interestingly, in this study it was observed that treatment with PqsBC inhibitors increased the 4-hydroxy-2-heptylquinoline-N-oxide (HQNO) levels. This molecular specie has been also linked to the emergence of antibiotic tolerance in *P. aeruginosa* (Hazan et al., 2016; Allegretta et al., 2017). HQNO appears to boost bacterial autolysis with the subsequent DNA release, which facilitates biofilm formation making the pathogen more tolerant to antibiotics (Hazan et al., 2016). Moreover, the HQNO produced by *P. aeruginosa* also influence the *S. aureus* susceptibility to antibiotics (Orazi and O'Toole, 2017; Radlinski et al., 2017). In another study, it was shown that *P. aeruginosa* Δ*pqsB* and Δ*pqsC* mutants that only produce DHQ, were more virulent in *C. elegans* than a quinolone-null mutant Δ*pqsAB*. An increased colonization capacity of *C. elegans* was observed for the Δ*pqsB* mutant in comparison with the Δ*pqsAB* mutant (Gruber et al., 2016).

Additional support for the feasibility of using QS signal biosynthesis inhibitors *in vivo* was demonstrated by the use of ambuic acid as an anti-virulence compound in a murine model of intradermal MRSA challenge. The treatment with ambuic acid impaired the virulence exerted by *S. aureus* in the infected animals, as it attenuated skin ulcer formation and the signs of infection-induced morbidity. The anti-virulence effect of ambuic acid was mediated by inhibition of the *agr* quorum sensing system (Todd et al., 2017).

### Inactivation of Quorum-Sensing Signal

Among the quorum quenching strategies one of the most exploited is the inactivation of QS signals (LaSarre and Federle, 2013). This is a strategy that exists in the natural interactions between microbial populations, and it has been extrapolated as an approach to modulating the virulence of bacterial pathogens. This virulence modulation through interference with QS signal is centered mainly on the use of QS signal-degrading enzymes (LaSarre and Federle, 2013; Fetzner, 2015). The advantage of this strategy is that it targets the QS signal after it is secreted to extracellular medium. Therefore, there is greater access to the target and challenges associated with penetrating bacterial cells are avoided. Moreover, as an extracellular factor is targeted, the emergence and spread of resistance could be less probable, but potential resistance mechanisms have been envisioned (Defoirdt et al., 2010; Fetzner, 2015; Vale et al., 2016).

The most thoroughly characterized quorum quenching enzymes are acyl-homoserine lactone (acyl-HSL) lactonases, acyl-HSL acylases, and acyl-HSL oxidoreductases, which target the QS signal acyl-homoserine lactones (acyl-HSLs) (Fetzner, 2015). The acyl-HSL lactonases and acyl-HSL acylases destroy acyl-HSL molecules via homoserine lactone ring hydrolysis (specifically the ester bond) or amide bond hydrolysis between the acyl tail and the homoserine lactone ring, respectively. Otherwise, the acyl-HSL oxidoreductases modify acyl-HSLs molecules chemically via oxidation or reduction of the acyl chain instead of degrading them (LaSarre and Federle, 2013; Fetzner, 2015). Other types of quorum quenching enzymes that have been characterized include *E. coli* LsrK kinase that targets the AI-2, and dioxygenases Hod from *Arthrobacter* sp. Rue61a, AqdC1 and AqdC2 from *Mycobacterium abscessus* subsp *abscessus* and *Rhodococcus erythropolis* BG43 that target alkylquinolone-type molecules (Pustelny et al., 2009; Roy et al., 2010; Müller et al., 2015; Birmes et al., 2017). LsrK catalyzes the phosphorylation of AI-2 molecules, rendering phospho-AI-2, whereas dioxygenases mediate a dioxygenolytic cleavage of PQS, rendering N-octanoylanthranilic acid and carbon monoxide (Pustelny et al., 2009; Roy et al., 2010).

The potential for using quorum quenching enzymes in clinical infection treatment is supported by several studies. Recently, Utari et al. (2018) used mouse models of pulmonary *P. aeruginosa* infection and showed the efficacy of intranasally administered PvdQ acylase in hindering *P. aeruginosa* virulence. In a lethal infection model, PvdQ-treated animals presented a 5-fold lower bacterial load than non-treated animals, as well as a longer survival time. Moreover, PvdQ-treated mice showed lower lung inflammation, CXCL2 and TNF-α levels than non-treated animals in a sub-lethal infection model. It is noteworthy that intranasally supplied PvdQ acylase was shown to be safe as it was well tolerated by animals (Utari et al., 2018). Previously, using a *Caenorhabditis elegans* infection model, the potential of PvdQ acylase as an anti-virulence agent had been shown (Papaioannou et al., 2009). Penicillin V acylases *Pa*PVA and *At*PVA from the Gram-negative bacteria *Pectobacterium atrosepticum* and *Agrobacterium tumefaciens* also exerted quorum quenching activity on *P. aeruginosa*. The supplementation of these two acylases to *P. aeruginosa* PAO1 provoked a reduction in 3-oxo-C_12_-HSL levels, elastase activity, pyocyanin and biofilm production. In addition, the survival rates of *G. mellonella* infected with *P. aeruginosa* PAO1 pre-treated with acylases were higher than *G. mellonella* larvae infected with *P. aeruginosa* PAO1 without acylases pre-treatment (Sunder et al., 2017).

Another quorum quenching enzyme that has been tested *in vivo* is the engineered lactonase *Sso*Pox-W263I. Using an acute lethal model of *P. aeruginosa* pneumonia in rats, Hraiech et al. (2014) demonstrated that intra-tracheally delivered *Sso*Pox-W263I immediately after infection with *P. aeruginosa* PAO1 significantly reduced the mortality rate and the lung damage. *Sso*Pox-W263I was well tolerated by rats (Hraiech et al., 2014). Recently, *Sso*Pox-W263I showed anti-virulence activity against clinical *P. aeruginosa* isolates. Interestingly, *Sso*Pox-W263I immobilization did not affect the anti-virulence activity against *P. aeruginosa* PAO1 (Guendouze et al., 2017). Moreover, AiiM lactonase attenuated *P. aeruginosa* PAO1 virulence in an acute pneumonia murine model. Mice infected via intratracheal with an AiiM-expressing *P. aeruginosa* PAO1 strain showed less lung injury, lower pro-inflammatory cytokines levels, and lower mortality than animals infected with an AiiM-nonexpressing *P. aeruginosa* PAO1 strain. In addition, in AiiM-expressing *P. aeruginosa* PAO1 infected mice there was a reduced systemic dissemination of the infection in comparison with the AiiM-nonexpressing *P. aeruginosa* PAO1 infected ones (Migiyama et al., 2013). Based on a *C. elegans* infection model it was demonstrated that lactonase MomL from *Muricauda olearia* increased the survival of *P. aeruginosa* PAO1-infected nematodes without showing toxic effects. However, a protective effect was not observed in *A. baumannii-*infected nematodes (Tang et al., 2015; Zhang et al., 2017b).

Furthermore, recently discovered quorum-quenching enzymes show potential as anti-virulence agents. Lactonases AaL isolated from *Alicyclobacillus acidoterrestris*, AiiK from *Kurthia huakui* LAM0618^T^ and Aii810 from Mao-tofu metagenome, inhibited virulence factors production and biofilm formation by *A. baumannii* and *P. aeruginosa* PAO1 without affecting bacterial growth (Fan et al., 2017; Bergonzi et al., 2018; Dong et al., 2018). Another newly described quorum-quenching enzyme is AidA, which was identified in *A. baumannii* clinical isolates (López et al., 2017).

In addition to quorum-quenching enzymes, QS signal inactivation is also reached by the action of anti-QS signal antibodies and synthetic polymers that sequester it (Piletska et al., 2010; LaSarre and Federle, 2013). The use of antibodies with therapeutic aims offers desirable effects, such as high specificity to the target coupled with low off-target cytotoxicity (Palliyil et al., 2014). However, developing anti-QS signal antibodies is a challenging task, because these signals are small size molecules and generally not structurally complex, making them poor antigens (Palliyil et al., 2014). Despite this, several studies support the potential of antibodies in disturbing quorum-sensing networks. Recently, centered on the *agr* type I quorum-sensing system of *S. aureus*, a virus-like particle (VLP)-based *agr* type I vaccine was developed using a *P. aeruginosa* RNA bacteriophage PP7 coat protein inserted with a *S. aureus* sequence-modified autoinducer peptide-1 (AIP1S, cysteine was substituted by serine, YST**S**DFIM). In this vaccinal candidate (PP7-AIP1S) the AIP1S peptide was exposed on the surface of the VLP, and immunized mice with PP7-AIP1S developed antibodies that specifically recognized the original *S. aureus* AIP1 *in vitro*. In addition, using a murine model of *S. aureus* SSTI (skin and soft tissue infection), it was observed that after a challenge with a virulent *S. aureus* USA300 isolate LAC (*agr*-type I), in PP7-AIP1S immunized mice reduced *agr*-type I-mediated pathogenesis was developed, compared to the non-immunized animals. Reduced alpha-hemolysin levels, as well as RNAIII transcription at the infection site in the immunized animals, together with the *in vitro* antibodies binding (from immunized animals) to AIP1 suggested the occurrence of immune suppression of *agr*-signaling during the infection (Daly et al., 2017). Using a peptide library displayed on VLP, O'Rourke et al. (2014) identified eight peptides (VLP-peptides) that bind specifically to the antigen-binding site of the monoclonal antibody AP4-24H11. This monoclonal antibody specifically bound and neutralized the autoinducer peptide-4 (AIP4) from *S. aureus* and protected animals from *S. aureus* pathogenicity, as was shown previously by Park et al. (2007). From the eight AP4-24H11-bonding VLP-peptides, two of them, when administered alone, apparently induced a protective response (reduced abscess and dermonecrosis) against *S. aureus agr*-type IV isolate AH1872 infection in immunized mice. Additionally, the immunization of mice with a combination of these two VLP-peptides protected those from *S. aureus* AH1872 infection via inhibition of *agr-*signaling (O'Rourke et al., 2014). Furthermore, it was observed that sheep-mouse chimeric monoclonal antibodies with affinity in the nanomolar range against HSL molecules protected *C. elegans* nematodes and mice infected with *P. aeruginosa* PA058. In infected mice, this protection appears to be associated with the antibody-mediated scavenging of HSL molecules and not by effects on the bacterial load (Palliyil et al., 2014). Moreover, immunized mice with 3-oxo- dodecanoyl homoserine lactone conjugated to BSA (3-oxo-c12-HSL-BSA) developed specific antibodies against the HSL and intermediate protection was observed after intranasal infection with *P. aeruginosa* PAO1. Interestingly, the lung bacterial burden was not affected in the immunized mice, and the levels of TNF-α (lung) and 3-oxo-c12-HSL (lung and serum) were lower than in non-immunized animals (Miyairi et al., 2006).

In addition to interference with QS signaling, monoclonal antibodies against QS signal molecules also protect from cytotoxic effects exerted by these molecules on host cells. Kaufmann et al. (2008) observed *in vitro* that the monoclonal antibody RS2-1G9 protected murine bone marrow-derived macrophages in a concentration-dependent fashion from the cytotoxicity associated with 3-oxo-C12-HSL (Kaufmann et al., 2008). Previously, it was demonstrated that serum from immunized animals with 3-oxo-C12-HSL-BSA inhibited the autoinducer-dependent apoptosis of the macrophage cell line P388D1 (Miyairi et al., 2006).

Synthetic polymers constitute another alternative for interference with QS signal. These polymers bind and sequester the QS signal without affecting bacterial growth; therefore, they should not exert selective pressure. A pioneering work by Piletska et al. (2010) demonstrated that signal-sequestering polymers interfered with the *Vibrio fischeri* QS network-based on 3-oxo-C6-HSL. The sequestering of 3-oxo-C6-HSL by the polymers impaired the bioluminescence production as well as biofilm formation (Piletska et al., 2010). In a subsequent work by this group, it was showed that an itaconic acid (IA)-based-molecular imprinted polymer (MIP) impaired *P. aeruginosa* biofilm formation by sequestering the 3-oxo-C12-HSL QS signal (Piletska et al., 2011). Moreover, linear polymers (IA-based polymers and methacrylic acid-based polymers) reduced *V. fischeri* bioluminescence and *Aeromonas hydrophila* biofilm production through lactones sequestering. The IA-based polymers were more effective than methacrylic acid-based polymers regarding the quorum-quenching activity. Importantly, the polymers did not show cytotoxic effects on mammalian cells and did not affect bacterial growth (Cavaleiro et al., 2015). Recently, it was observed that 2-hydroxyethyl methacrylate (HEMA)-based MIPs suppressed the biofilm formation by *P. aeruginosa*; however, IA-based MIPs were not effective in the biofilm attenuation (Ma et al., 2018).

### Interference With Quorum Sensing Signal Detection

Interference with signal detection is another of the most exploited strategies for disrupting QS systems. Some of these QS signal detection inhibitors are signal structural analogs that compete with the signal molecule by binding at the ligand-binding site in the receptor (Stevens et al., 2010). Moreover, other inhibitors could act in a non-competitive fashion (e.g., halogenate furanones, isothiocyanate-based covalent inhibitors, and flavonoids) (Koch et al., 2005; Amara et al., 2016; Paczkowski et al., 2017). The inhibitors binding to the receptors directly affect the signaling cascade by different pathways, including block signal binding, structural destabilization of the receptor, impaired receptor dimerization, impaired DNA binding, or impaired interaction with RNA polymerase (Stevens et al., 2010; Paczkowski et al., 2017; Suneby et al., 2017). Moreover, it has been shown that agonists of the QS signal can also exert inhibitory activity on QS systems. Some QS circuits are arranged in hierarchically cross-regulated networks, e.g., in *P. aeruginosa* the *las*-QS system positively regulates *rhl*- and *pqs*-QS systems, in addition the activated *pqs*-QS system also positively regulates the *rhl*-QS system, whereas this exerts negative regulation on the *pqs*-QS system (Lee and Zhang, 2015). Therefore, by modulating the activity of one QS system, it is possible to influence the activity of the other QS systems. In this respect, Welsh et al. (2015) observed that some agonists of RhlR attenuated the expression of virulence factors controlled by the *pqs*-QS system in *P. aeruginosa*. Disruption of cross-regulation of *rhl*-*pqs* systems was proposed as a novel mechanism of QS inhibition (Welsh et al., 2015).

The use of small molecules to disturb QS signal detection has to face several challenges. In this respect, structural stability of the inhibitors is a very important issue; some structural analogs of HSLs and AIPs are prone to hydrolysis, depending on the characteristics of the media (Glansdorp et al., 2004; Vasquez et al., 2017). In addition, inhibitors could be potentially degraded by enzymes as well as targeted by efflux pumps (Maeda et al., 2012; Grandclément et al., 2015). Moreover, the inhibitory effect observed could be strain-dependent, and it is therefore important to include several strains in the studies (García-Contreras et al., 2015). Despite the challenges, the feasibility of QS signal detection inhibition as a quorum-quenching strategy is supported by several *in vivo* studies.

Meta-bromo-thiolactone (mBTL) is a partial agonist/ partial antagonist of both RhlR and LasR receptors in the HSL-guided QS systems of *P. aeruginosa*, and RhlR inhibition is its main mechanism of action *in vivo*. This compound potently inhibited *P. aeruginosa* PA14 pyocyanin and biofilm production without affecting bacterial growth. In addition, the treatment of *P. aeruginosa* PA14 with mBTL down-regulated the expression of several LasR- and RhlR-controlled virulence factor genes. The treatment of wild-type and *P. aeruginosa* PA14 *lasR* mutant strains with mBTL reduced the pathogenesis exerted by these strains in *C. elegans* and human lung carcinoma cell line A549 (O'Loughlin et al., 2013). Another inhibitor of *P. aeruginosa* HSL-based QS systems that has been tested *in vivo* is the fungal metabolite terrein. The treatment of *P. aeruginosa* PAO1 with terrein provoked a reduction in a dose-dependent manner in the production of virulence factors elastase, pyocyanin, and rhamnolipid as well as in biofilm formation without affecting bacterial growth. In addition, terrein showed to be more stable than the QS inhibitor furanone C-30 and enhanced the anti-biofilm activity of ciprofloxacin when used in combination. Importantly, terrein mediated protection of *C. elegans* and mice against *P. aeruginosa* PAO1infection in a fast killing infection assay and murine airway infection model, respectively. Interestingly, it was observed that the QS system and c-di-GMP signaling pathway could be interconnected, and that terrein could act as a dual inhibitor of these systems (Kim et al., 2018). Moreover, HSL analogs that act as covalent inhibitors of LasR receptor were seen to be promising *in vivo* tests. Specifically, the isothiocyanate- and fluoroisothiocyanate-based covalent inhibitors (ITC-12 and ITC-F, respectively) attenuated the virulence of *P. aeruginosa* PAO1-UW and consequently increased the survival of *C. elegans* worms during an infection assay with this pathogen. The ITC-F treated group showed a significant survival rate in comparison with the control group. Moreover, using an *ex-vivo* human skin burn wound model it was observed that ITC-F and ITC-12 treatment impaired the establishment of infection by *P. aeruginosa* PA14 (Amara et al., 2016). In addition to HSL-based QS systems in *P. aeruginosa*, the PQS-based QS system is also involved in the regulation of virulence factor production. In this regard, maybe inhibitors that could affect these two QS system types would be desirable. Among them, 3-Phenyllactic acid (PLA) is an organic compound produced by *Lactobacillus* spp that acts as a QS sensing inhibitor that potentially could bind to RhlR and PqsR receptors with high affinity (Chatterjee et al., 2017). Recently, Chatterjee et al. (2017) showed that PLA impaired the attachment of *P. aeruginosa* PAO1 on a catheter tube, using a Medaka fish intraperitoneal catheter-associated infection models (Chatterjee et al., 2017).

Furthermore, *agr*-QS system inhibition has been shown to be an achievable strategy for controlling the virulence of pathogens like *S. aureus*. In this regard, atopic dermatitis is a chronic inflammatory skin disease where *S. aureus* triggers an immunopathology response through mast cell degranulation. This mast cell degranulation could be induced by the bacterial δ-toxin, which is encoded by the *hld* gene that is under control of the *agr-*QS system (Baldry et al., 2018; Geoghegan et al., 2018). Recently, the effectiveness of the *agr*-QS inhibitor solonamide B in suppressing the *S. aureus* δ-toxin-induced-inflammatory response was tested using a modified epicutaneous colonization mouse model. Animals infected with *S. aureus* and treated with solonamide B showed a reduced skin inflammatory cell infiltrate, less skin damage, reduced RNAIII expression and production of pro-inflammatory cytokines in comparison to non-treated animals, suggesting that *agr*-QS inhibitors could effectively attenuate *S. aureus* pathogenesis *in vivo* (Baldry et al., 2018).

### Innovative Quorum Quenching Strategies

In addition to the use of quorum quenching enzymes and quorum sensing inhibitors, some innovative therapeutic strategies to interfere with quorum sensing networks are being developed. One of the challenges in disrupting quorum sensing networks is the fact that a pathogen may possess several QS systems of the same class, for example *P. aeruginosa* contains the AHL-based systems LasRI and RhlRI (Lee and Zhang, 2015). Therefore, acquiring complete inhibition of the QS systems using quorum-quenching enzymes or quorum-quenching inhibitors in a monotherapy-based scheme could be difficult (Fong et al., 2018). Based on this challenge, Fong et al. (2018) tested the capacity of a combinatory therapy using the quorum-quenching enzyme AiiA and the quorum-sensing inhibitor G1 (LuxR-type receptor inhibitor) to suppress the QS systems in *P. aeruginosa*. It was observed that combinatory therapy inhibited the expression of *lasB-gfp, pqsA-gfp*, and *rhlA-gfp* in *P. aeruginosa* PAO1 bioreporter strains more potently than single treatments. The *rhlA* gene is involved in the biosynthesis of rhamnolipid and is under RhlR-transcriptional regulation. In accordance with this, the inhibitory effect on rhamnolipid biosynthesis was verified in a *P. aeruginosa* PAO1 strain. The level of synthetized rhamnolipid in the combinatory therapy-treated bacteria was nearly to the rhamnolipid level in a Δ*lasI*Δ*rhlI* mutant strain (Fong et al., 2018). All this evidence suggests that using combinatory therapy with different types of quorum-quenching agents it is possible to disturb diverse quorum-sensing systems existing in pathogens.

In the combinatory therapy described above, a multi-target effect is achieved by joining two therapeutic agents that target different components in the QS networks. However, it is possible to get the same multi-target effect using a single compound (Thomann et al., 2016; Maura et al., 2017). Recently a drug with dual inhibitory activity toward the PqsR and PqsD components of the *P. aeruginosa pqs* QS system was developed. This dual inhibitor [2-(methylsulfonyl)-4-(1H-tetrazol-1-yl)pyrimidine] was developed from a common molecular scaffold existing in single PqsR- antagonist and PqsD-inhibitor. *In vitro* analysis showed that the dual inhibitor disturbed the production of the virulence factors pyocyanin and pyoverdine as well as the biofilm production by *P. aeruginosa* PA14. In addition, the dual inhibitory compound increased the survival rate of *G. mellonella* larvae infected with *P. aeruginosa* PA14 (Thomann et al., 2016). Moreover, Maura et al. (2017) observed that some benzamide-benzimidazole-based compounds also act as dual inhibitors of the *pqs*-QS system in *P. aeruginosa*. These dual inhibitory compounds targeted the proteins PqsR (MvfR) and PqsBC and could be grouped depending on their inhibition patterns in: PqsR-PqsBC dual inhibitors with high anti-PqsR and high anti-PqsBC activity or PqsR-PqsBC dual inhibitors with low anti-PqsR activity and high anti-PqsBC activity. The treatment with some of these dual inhibitors increased the survival rate of human lung epithelial cells and RAW264.7 macrophages when infected with *P. aeruginosa* PA14 (Maura et al., 2017). Among the dual inhibitors, those exert a high anti-PqsR activity constitute an attractive therapeutic option because interfere with the production of 2-AA and consequently limit the emergence of antibiotic-tolerant bacteria as was previously discussed.

Recently it has been demonstrated that it is possible to manipulate the AI-2 levels through “controller cells” (Quan et al., 2017). These “controller cells” are based on a subset of “consumer cells” and another of “supplier cells.” The “consumer cells” were engineered to overexpress genes involved in the uptake and processing of AI-2 in *E. coli* (e.g., *lsrACDBK* and *lsrACDBFGK*) while the “supplier cells” in genes involved in the biosynthesis of AI-2 (*luxS* and *mtn*). Because these “controller cells” influence the environmental AI-2 levels they will have a direct impact on biofilm formation. In line with this, it was observed that “consumer cells” decreased biofilm formation by *E. coli* reporter strain, whereas “supplier cells” enhanced biofilm formation (Quan et al., 2017). This suggests a route toward future therapeutic strategies based on engineered cells that act as “controller cells.” In addition, it is possible to modulate microbial behavior through AI-2 levels via a synthetic mammalian cell-based microbial-control device, as was demonstrated by Sedlmayer et al. (2018). This microbial-control device consisted of engineered mammalian cells with a formyl peptide sensor module coupled to AI-2 production and release module. Essentially, the engineered mammalian cells detect formyl peptides released by pathogens (peptides produced by a broad range of bacterial species) and trigger the production and release of AI-2. It was showed that biofilm formation by *Candida albicans* was reduced when this pathogen was co-cultured with microbial-control-engineered cells. This system appears to be a promising anti-virulence strategy, as ubiquitous pathogen signals are detected with high sensitivity (nM range), and robust production of the autoinducer takes place (without being toxic for the host) influencing bacterial communication without exerting selective pressure. In addition, the fact that autoinducer production is coupled to signal detection allows a synchronized response in accordance with the infection dynamic (Sedlmayer et al., 2018). Moreover, it possible to engineer bacteria that will sense the presence of pathogenic bacteria via the quorum sensing system and, once detected, will release anti-pathogens agents. Hwang et al. (2017) engineered a probiotic *E. coli* Nissle 1917 strain for sense 3-oxo-C12 HSL from *P. aeruginosa* and respond by autolysing itself via lysin E7 with the consequent release of the bacteriocin pyocin S5 and the anti-biofilm enzyme DspB, which exerted an anti-*P. aeruginosa* activity. The feasibility of this approach was demonstrated *in vivo* using *C. elegans* and murine infection models, where the engineered strain showed prophylactic and therapeutic effects (Hwang et al., 2017).

Although the strategy of interference with autoinducer biosynthesis has been based on the discovery and design of small molecules that inhibit the enzymatic activity, it has been envisioned that engineered bacterial strains could be an alternative. Recently, it was showed that it is possible to disrupt biofilm production in clinical isolates through the manipulation of the expression levels of the enzyme LuxS. Specifically, using the Clustered Regularly Interspaced Short Palindromic Repeats-Cas 9 interference (CRISPRi) system, the expression of LuxS enzyme was suppressed in clinical *E. coli* isolates. It was suggested that CRISPRi edited cells could be an alternative strategy for controlling biofilm production in nosocomial settings through CRISPRi system delivery via nucleic acid conjugation (Zuberi et al., 2017). However, delivery of CRISPRi system in nosocomial setting via nucleic acid conjugation could be a very challenging task. Given the fact that nucleic acid conjugation could occur between different bacterial species (Musovic et al., 2006; Goren et al., 2010; Crémet et al., 2012; Van Meervenne et al., 2012); could be possible the transfer of the CRISPRi system from the edited cells to co-existing bacteria other than target bacteria. In this regard, maybe the utilization of narrow host range plasmids as vectors for CRISPRi delivery could limit such potential off-target effect. In addition, the nucleic acid conjugation effectivity in established biofilms could be compromised (Merkey et al., 2011; Stalder and Top, 2016). This could limit the use of CRISPRi edited cells for the treatment of formed biofilms. In this sense, maybe the utilization of engineered phages as vehicles for CRISPRi system delivery could be an attractive alternative. Phages have shown be a CRISPR delivery system with specificity to pathogenic bacteria as well as with capacity to removing established biofilms (Lu and Collins, 2007; Bikard et al., 2014; Citorik et al., 2014; Alves et al., 2016; Fong et al., 2017).

## Preventing Biofilm Formation and Affecting the Biofilm Structure Without Killing Bacteria

Bacteria can live in a community called biofilm, a structure that can be formed by extracellular polymeric substances, such as DNA, protein, and polysaccharides (Flemming et al., 2016). After forming biofilms, bacteria can disperse and colonize other environments (Fleming and Rumbaugh, 2017). This lifestyle can protect bacteria against potential environmental stress, such as antibiotics and host defense components (Hall and Mah, 2017; Tseng et al., 2018). In the clinical situation, biofilm formed by pathogenic bacteria can establish themselves on human surfaces or medical devices, including implants, catheter, endotracheal tubes and others (Rieger et al., 2016; Konstantinović et al., 2017; Kenaley et al., 2018; Silva et al., 2018). Biofilm formation on these surfaces can serve as a source of infection. The successful establishment of pathogenic biofilm on human surfaces can cause chronic infections and limit the success of antibiotic therapy (Rybtke et al., 2015; Li et al., 2017). In general, combating biofilms may require high antibiotic doses and a combination of strategies (Ribeiro et al., 2016). Unfortunately, many of the marketed antibiotics fail to affect biofilm, especially if they are formed by resistant bacteria. To overcome this problem, researchers have prospected compounds from the natural world (from animals, plants, fungi, viruses, and even bacteria) or synthetics (synthesized through the chemical process and/or screened from chemical libraries) (Rajput et al., 2017). In both situations, anti-biofilm agents can be represented by a variety of organic and inorganic chemical compounds (Rajput et al., 2017).

Anti-virulence compounds against biofilms could be used to limit bacterial adhesion on surfaces (Liu et al., 2018; Ranfaing et al., 2018) to affect the production of an extracellular matrix (Feng et al., 2018) and to disturb the existing biofilm (Puga et al., 2018; Table 1). Some examples of anti-virulence compounds cited here work against non-pathogenic bacteria to humans and animals. However, the approaches using these bacteria may serve as proof of principle to study anti-virulence compounds against biofilms in a general way.

In the prevention scenario, one possibility consists of interfering with structures associated with the successful establishment of biofilms such as flagella (that favor the bacterial motility and interaction with surfaces) and fimbriae (with structures that facilitate bacterial adhesion). Higrocin C (a compound isolated from marine-derived *Streptomyces* sp. SCSGAA 0027) for example, suppressed swimming motility of *Bacillus amyloliquefaciens* SCSGAB0082, which could explain the biofilm inhibition (Wang et al., 2018a). Transcriptome studies showed downregulation (more than twofold) of genes associated with bacterial chemotaxis and flagellar motor (Wang et al., 2018a). Coumarin, for example, presents the ability to prevent bacteria biofilm without affecting bacterial growth (Lee et al., 2014). This compound repressed curli genes and motility genes in *E. coli* O157:H7 and reduced fimbriae production, swarming motility, and biofilm formation (Lee et al., 2014).

Another way to prevent biofilm formation consists of affecting the extracellular matrix production. A chemical compound named TCC (3, 3′, 4′, 5-tetrachlorosalicylanilide) for example, inhibited *B. subtilis* biofilm formation by reducing extracellular matrix production. This was associated with the repression of SinR protein negative regulated genes (involved in extracellular matrix production) (Feng et al., 2018). Other studies have shown that compounds that prevent biofilm formation can potentially affect bacterial cell communication by degrading quorum-sensing molecules (Ivanova et al., 2015; Passos da Silva et al., 2017).

In the context of combating existing biofilms, compounds can be used to destroy components of extracellular matrix, such DNA, proteins and carbohydrates (Puga et al., 2018). In this context, enzymes have been used as potential agents to disrupt mono and polymicrobial biofilm (Puga et al., 2018). DNAse I, for example, presents the ability to degrade extracellular DNA of *Campylobacter jejuni*, promoting biofilm removal without affecting bacterial viability (Brown et al., 2015).

The understanding of mechanisms involved in the formation of bacterial biofilm, as well as the understanding of their cells and biofilm structures, could indicate possible targets to develop compounds that affect biofilm without killing bacteria. In addition to potential anti-biofilm therapy, agents that can prevent or disperse biofilm could potentially combine with anti-virulence compounds. For example, anti-virulence agents could be used to neutralize endotoxins from bacterial cells that disperse from biofilms and thus prevent or minimize the harmful effects of the host inflammatory response against bacterial infection.

## Bacterial Toxin Neutralization

It is known that pathogenic bacteria may produce diverse virulence determinants in order to successfully survive host system responses, as well as colonizing a host (Kong et al., 2016). Among them, toxins comprise proteins expressed by bacteria during post-exponential and early stationary phases that have been divided into different classes, including hemolysin (Powers et al., 2015), leukotoxin (Zivkovic et al., 2011), exfoliative toxins (Bukowski et al., 2010), endotoxin (Heinbockel et al., 2018), among others. These protein-based toxins are intrinsically related to physical damage, biochemical degradation and signaling interruption in the host cells, resulting in immune system evasion and characterizing pathogen-to-host interactions (Wei et al., 2017). Bacterial toxin neutralization, for instance, has been shown to compromise bacterial proliferation and survival in the host (Ortines et al., 2018). More importantly, unlike antibiotic-based treatments, anti-toxin or anti-virulence therapies do not affect bacterial viability directly and, as a consequence, could impose reduced selective pressure, probably decreasing the frequency of resistance events (Rasko and Sperandio, 2010). In addition, anti-virulence compounds are also known to preserve the host's endogenous microbiome as they target virulent factors secreted exclusively by pathogenic bacteria (Clatworthy et al., 2007). In this context, here we described compounds, including antibodies, nanoparticles, small molecules, and bioactive peptides (Figure 1 and Table 1), which have been studied recently as promising candidates for anti-virulence therapies that aim to treat and prevent bacterial infections.

The α-toxin (AT), also known as α-hemolysin, is a key virulence factor expressed by *S. aureus* that has been investigated in different animal infection models, including bacteremia, pneumonia and skin/soft tissue infections (Surewaard et al., 2018). This toxin is capable of lysing red blood cells, and also targets monocytes, macrophages and neutrophils (Bubeck Wardenburg et al., 2007). Moreover, in the clinic, AT levels in patients are often correlated with disease severity (Jenkins et al., 2015). Studies have shown that rabbits with acute bacterial skin and skin structure infections (ABSSSI) caused by AT-expressing methicillin-resistant *S. aureus* (MRSA) develop severe infections similar to those observed in humans, including the presence of large dermonecrotic lesions. In contrast, rabbits infected with a mutant deficient AT strain developed only small dermonecrotic lesions (Le et al., 2016). One major anti-virulence strategy to neutralize AT consists of using antibodies. The study cited above also reported a significant decrease in the disease severity thought AT neutralization by treating the rabbits with an anti-AT human monoclonal antibody (mAb) (MEDI4893^*^) (Le et al., 2016). Similarly, Ortines et al. (2018) observed in non-diabetic and diabetic mice that *S. aureus*-infected animals passively immunized with anti-AT mAb (MEDI4893^*^) showed decreased wound size and bacterial counts when compared to the untreated controls. Moreover, those authors also showed the differential host immune response effects, revealing different patterns of macrophage, monocyte and neutrophil infiltrates, as well as neutrophil extracellular traps (NETs) in non-diabetic and diabetic mice (Ortines et al., 2018).

In addition to skin infections, *S. aureus* strains are often associated with respiratory mono-infections and co-infections with Gram-negative strains, including *P. aeruginosa* and *Klebsiella pneumoniae*. In a study by Cohen et al. (2016), it was shown that *S. aureus* AT, in a mixed pathogen-lung infection model, could potentiate Gram-negative bacterial dissemination and lethality. This situation, however, could be circumvented by the passive immunization of mice with an anti-AT mAb, leading to *S. aureus* and co-pathogens (Gram-negative bacteria) clearance in the lungs (Cohen et al., 2016). Additionally to mAb, the intravenous immunoglobulin (IVIG), which consists of a polyclonal human antibody pool, has been investigated regarding its protective effects against necrotizing pneumonia caused by different epidemic community-associated and hospital-associated MRSA strains (Diep et al., 2016). As reported by Diep et al. (2016), two IVIG antibodies specific to an AT (α-hemolysin, HTa) and a Panton-Valentine leukocidin (PVL) conferred protection on immunized rabbits against MRSA, leading to improved survival outcomes (Diep et al., 2016).

In the clinic, patients affected by bacteremia, including *S. aureus*, may present occlusion of small blood vessels by the formation of large platelet aggregates (van der Poll and Opal, 2008). In a recent study, it was reported that AT induces rapid platelet aggregation and liver injury, causing multi-organ dysfunction during *S. aureus* sepsis (Surewaard et al., 2018). Interestingly, however, all these damaging effects could be prevented in mice treated with the anti-AT mAb (MEDI4893^*^) (Surewaard et al., 2018), thus reinforcing the importance of monoclonal antibodies as bacterial toxin neutralizing agents in anti-virulence therapies. More recently, Wang et al. (2018b), reported a novel vaccine platform based on extracellular vesicles (EVs) from *S. aureus*. In that work, the authors purified EVs from a genetically engineered *S. aureus* capable of overexpressing detoxified cytolysins (HlaH35L and LuKE), which were non-toxic, immunogenic and protected mice from lethal sepsis caused by *S. aureus* (Wang et al., 2018b). Also in the field of *S. aureus* toxin neutralization, the monoclonal antibody, ASN100 (Arsanis Inc.), which consists of the combination of two human IgG1k monoclonal antibodies, ASN1 and ASN2, has shown promising results in the neutralization of six *S. aureus* toxins (Rouha et al., 2015; Badarau et al., 2016). Despite the advances in the usage ASN100 in the clinic, the company Arsanis Inc. has discontinued a phase II clinical trial for ASN100 as it failed to prove its effectiveness in high-risk, mechanically ventilated patients with *S. aureus* pneumonia.

Antibodies have also been applied as anti-virulent therapies involving *Clostridium difficile*, which represents a primary cause of nosocomial antibiotic-related diarrhea. This bacterium produces two main virulence factors, toxin A (TcdA) and toxin B (TcdB), responsible for gastrointestinal epithelial damage and colonic inflammation. In this matter, the engineering and use of TcdA/B-neutralizing antibodies appears as a promising approach to counter diarrhea episodes caused by *C. difficile* infections. With that in mind, Andersen et al. (2016) developed an antitoxin strategy to express TcdB-neutralizing antibody fragments in *Lactobacillus* strains in the gastrointestinal tract of hamsters infected with a TcdA^−^/ TcdB^+^ *C. difficile* strain. Initially, *in vitro* studies were carried out to confirm the ability of the expressed fragments in neutralizing the cytotoxic effect of TcdB. Moreover, *in vivo* assays revealed that *Lactobacillus* strains expressing two TcdB-neutralizing antibodies led to improved survival rates in the treated group. Furthermore, the protection with TcdB-neutralizing antibodies also preserved the gastrointestinal tract of the animals as no damages or limited inflammation were observed (Andersen et al., 2016). In addition to antibody-based therapies, studies have also explored the potential of small molecules as inhibitors of *C. difficile* TcdB. Tam et al. (2015) reported a high-throughput phenotypic method for screening small molecules capable of protecting human cells from TcdB. As a result, the authors reported a series of small molecules with diverse mechanisms of action on TcdB, including direct binding, sequestration of TcdB, non-competitive inhibition of the glucosyl-transferase activity of TcdB, as well as endosomal maturation inhibition (Tam et al., 2015). However, *in vivo* studies are still underway to confirm the effectiveness of these small molecules in *C. difficile* infections.

Apart from the application of anti-toxin antibodies and small molecules in anti-virulence therapies, studies have also highlighted the importance of engineered nanoparticle mimicking cell membranes (e.g., liposomes) in sequestering cytotoxic bacterial toxins both *in vitro* and *in vivo* (Fang et al., 2015). Artificial liposomes are constituted exclusively of natural lipids and therefore are not active against bacteria, thus allowing their usage in combination with antibiotics for bacterial infection treatment. Henry et al. (2015), for instance, showed the potential of artificial liposomes in sequestering bacterial toxins *in vitro*, along with the preservation of the integrity of mammalian cells. The authors also observed that, during *in vivo* experiments, the administration of artificial liposomes resulted in mice recovering from septicemia caused by *S. aureus* and *Streptococcus pneumoniae*, as well as mice being protected against pneumonia (Henry et al., 2015). Moreover, combining the artificial liposomes with conventional antibiotics, including vancomycin and penicillin, improved survival rates were observed when compared to mono-therapies (Henry et al., 2015).

Bacteria secrete a wide variety of toxins during host colonization and infection, which represents a bottleneck when it comes to vaccine development aiming at anti-virulence therapies. Indeed, vaccine strategies based on multiple targets (bacterial toxins) have already been reported; however, the identification and further confirmation of virulence factors secreted by bacteria is considered a costly and time-consuming method (Fujita and Taguchi, 2011). As an alternative, studies have proposed the use of multiantigenic nanotoxoids based on naturally occurring bacterial proteins to develop vaccines against pathogenic bacteria. Wei et al. (2017) reported a feasible approach for entrapping diverse toxins from bacterial protein preparations using a membrane-coated nanosponge construct capable of delivering these virulence factors in the organism and, consequently, combating bacterial infections. As for the other anti-virulence therapies here described, the nanoparticle-based neutralization and delivery not only usefully prevent severe bacterial infections but also decrease the risk of antibiotic resistance events (Wei et al., 2017).

Besides the secretion of protein-based toxins, the bacterial LPS in the host's blood stream is known to cause severe immune system stimulation, resulting in septic shock and sepsis (Rietschel et al., 1996). Among the strategies to neutralize LPS, the application of antimicrobial peptides (AMPs) has shown promising results. Moreover, the mechanisms of action and structural arrangements of some AMPs in contact with LPS have already been investigated, including polymyxins (Pristovsek and Kidric, 1999), temporins (Bhunia et al., 2011), and melittins (Bhunia et al., 2007). This class of antimicrobials is well known for its multifunctionality and structural diversity. Studies have shown that AMPs with extended activities, including immunomodulatory, are capable of binding to LPS and, consequently, decreasing the production of nitric oxide and tumor necrosis factor-α (TNF-α), which are commonly related to tissue damage (Pulido et al., 2012). Chih et al. (2015), for instance, have reported the antiendotoxin effects of two antimicrobial peptides, S1 and KWWK. Interestingly, the authors observed that LPS-neutralizing activities were directly related to the addition of β-naphthylalanine end-tags in both peptides, which was also reflected in the dose-dependent inhibition of nitrite oxide production and TNF-α release *in vitro* and *in vivo* (Chih et al., 2015). In addition, other AMPs, including members from the Pep19-2.5 family (Heinbockel et al., 2018) and retrocyclins (Kudryashova et al., 2015), have revealed the ability to unfold bacterial toxins, as well as causing conformational changes such as toxin aggregation and fluidity (Heinbockel et al., 2018).

## Future Directions

The antimicrobial resistance threat has driven the global scientific community to search for effective solutions. Given the fact that antimicrobial resistance is a multifactorial phenomenon, the solution for this problem involves a range of approaches focused on controlling the factors that facilitate the emergence and spread of resistance. One of these approaches consists of developing new therapeutic agents that operate under different principles to the currently available antibiotics. In this respect, anti-virulence therapy has been envisioned as a promising alternative with the aim of controlling pathogen virulence in a pathogen-specific fashion, without exerting strong selective pressure on the pathogens.

However, as an emerging therapeutic strategy, anti-virulence therapy has to face several challenges. The selection of the targeted virulence factor(s) is of critical importance for the effectiveness of the strategy in terms of evolutionary robustness. In line with this, a suitable target should be a virulence factor whose disruption does not imply (or imply minimal) fitness consequences for the pathogen (Vale et al., 2016). Moreover, a virulence factor that is conserved between different pathogens could be ideal, because in principle it would be possible to treat polymicrobial infections with a single anti-virulence drug (Maura et al., 2016). It is necessary to understand the detailed dynamics of action of the targeted virulence factor as well as the dynamics of production (Dickey et al., 2017). For example, during *P. aeruginosa* infection of cystic fibrosis patients take place an acute to chronic infection transition. This shift involves down-regulation of virulence factors as the flagellum, T3SS secretion system, proteases, and others; while virulence factors as exopolysaccharides are up-regulated (Hogardt and Heesemann, 2013; Sousa and Pereira, 2014). Therefore, the anti-virulence agent that target some of these virulence factors should be supplemented in accordance with this dynamic of expression. In addition to knowing the targeted virulence factor production dynamics, it is important to know if this virulence factor undergoes chemical modifications that modulate its activity. Furthermore, as anti-virulence therapy works in a pathogen-specific fashion, it is important to have diagnostic methods like matrix-assisted laser desorption ionization-time of flight mass spectrometry (MALDI-TOF), microarray-based nucleic acid test, magnetic resonance-based diagnostic, fluorescence *in situ* hybridization (FISH) test, next generation sequencing (NGS) and multiplex PCR-based diagnostic test, which permit rapid and precise identification of the infection-causing pathogen (Dickey et al., 2017; Messacar et al., 2017). It is also mandatory to define which parameters will be taken into account for measuring the effectivity of the anti-virulence therapy and for which type of infection the therapy is most suitable (Maura et al., 2016; Dickey et al., 2017). For example, in certain types of *S. aureus* infections (e.g., chronic and bacteremia), a dysfunctional *agr*-QS system appears to be beneficial for the pathogen (Khan et al., 2015). Moreover, recently it has been reported that defective *agr*-QS system could mediate the tolerance to certain antibiotics (gentamicin and ciprofloxacin; Kumar et al., 2017). In addition, it has been suggested that phenol-soluble modulin toxins (PSMs) are involved in the control of *S. aureus* persister cells population (Bojer et al., 2018). Because the PSMs production is under the control of the *agr*-QS system, it is probably that a defective *agr*-QS system down-regulate the expression of PSMs which could favor the emergence of persister cells to certain antibiotics. Therefore, in the above-pointed situations maybe anti-virulence therapies based on *agr*-QS system inhibition could be not a feasible strategy. Although anti-virulence therapy is an emerging field, several potential anti-virulence drugs have already been identified, and existing chemical libraries for antibiotic discovery could be a valuable source for rapid identification of novel anti-virulence drugs (Maura et al., 2016; Dickey et al., 2017). At this point, it is necessary to direct these potential anti-virulence candidates toward pre-clinical and clinical trials.

## Author Contributions

OF, MC, and SR wrote the manuscript. MC performed the figures. OLF designed and revised the manuscript.

### Conflict of Interest Statement

The authors declare that the research was conducted in the absence of any commercial or financial relationships that could be construed as a potential conflict of interest.
